# Review: Shifts of rumen microbiota by feeding non-fibrous carbohydrates to improve cattle performance

**DOI:** 10.3389/fmicb.2026.1735296

**Published:** 2026-02-26

**Authors:** Johnny M. Souza, Pedro H. C. Ribeiro, Danilo D. Millen

**Affiliations:** Department of Animal Science, School of Agricultural and Veterinary Sciences, São Paulo State University (UNESP), Jaboticabal, São Paulo, Brazil

**Keywords:** cross-feeding, fermentation, forage-to-concentrate ratio, microorganisms, ruminants

## Abstract

Ruminants play an essential role in food production due to their ability to utilize forages through fermentation in the rumen. This fermentative chamber hosts a diverse microbial community capable of degrading fiber and non-fiber carbohydrates, producing short-chain fatty acids (SCFAs) and microbial protein, which are essential for the animal’s metabolism. Throughout their evolution, ruminants developed a symbiotic relationship with microorganisms specialized in the degradation of plant fibers, enabling the use of forages as a dietary foundation. However, modern intensive production systems have introduced concentrate ingredients to their diets (such as grains and industrial by-products), which represent a significant departure from ancestral diets based exclusively on forages. Dietary composition is the primary factor driving changes in the ruminal microbiota and can significantly alter its composition. Variations in the forage-to-concentrate ratio can drastically alter microbial activity, affecting the stability of the ruminal ecosystem. Sequencing technologies and omics approaches have enhanced the understanding of this ecology, allowing for more effective nutritional interventions. The objective of this review is to assess how contemporary diets in intensive production systems differ from ancestral, forage-only diets and how these differences reshape the ruminal microbiota. To this end, we characterized the variations in the ruminal microbiota composition of animals fed high-concentrate and high-forage diets, describing the specific microbial profiles of each condition and identifying beneficial and potentially detrimental microorganisms. This review synthesizes current evidence on how dietary transitions reshape ruminal microbial cross-feeding networks and proposes an integrative framework linking microbial symbiotic balance, rumen health, and production efficiency. By emphasizing the dynamic regulation of microbial interactions rather than isolated taxa, this work highlights cross-feeding stability as a central target for nutritional, microbial, and genetic interventions in intensive ruminant production systems.

## 1 Introduction

The evolution of ruminants over millions of years resulted in the development of a highly specialized digestive system capable of extracting energy from fibrous plant materials through a complex ruminal microbial community. This symbiotic relationship allowed ruminants to occupy unique ecological niches, converting forages, indigestible to other mammals, into high nutritional value products such as meat and milk (Van Soest, 1994). However, modern intensive production systems have introduced a paradigmatic shift in the diets of these animals, incorporating high proportions of concentrate ingredients that differ drastically from the forages that shaped their evolution.

The rumen, one of the stomach compartments of ruminants, is considered a natural bioreactor that houses a complex and highly specialized microbial community. The ruminal microbiota maintains a symbiotic relationship with the host and facilitates the utilization of indigestible plant materials; however, its structure is profoundly altered in response to changes in diet composition. Like other mammals, ruminants do not produce cellulolytic and hemicellulolytic enzymes to digest plant compounds; instead, they depend entirely on rumen microorganisms for this function (McCann et al., 2014; Gruninger et al., 2019; Shen et al., 2019).

Based on this fact, modern diets appear to overlook the rumen microbiota’s ability to degrade and ferment fiber. However, bacterial communities that ferment fiber components remain in the rumen of animals fed high-concentrate diets. Therefore, the central question that emerges is: how distant are the diets used in current intensive production systems from ancestral diets based exclusively on forages? This question is fundamental to understanding the impacts of productive intensification on ruminal health and animal welfare. Studies in beef and dairy cattle demonstrate that the transition from forage-based diets to concentrate-rich diets results in profound alterations in the ruminal microbiota, with significant implications for animal health and productivity (Petri et al., 2013; Plaizier et al., 2017).

The ruminal microbiota, composed of trillions of microorganisms divided into two groups of Prokaryotes (bacteria and archaea), two groups of Eukaryotes (protozoa and fungi), and a vast population of viruses, predominantly bacteriophages, is fundamental for the digestion of complex nutrients present in the diet (Liu et al., 2022; Lobo and Faciola, 2021). As the main end products of different metabolic pathways, the entire ruminal microbiota produces short-chain fatty acids (SCFAs), microbial protein, heat, water, ATP, carbon dioxide, and methane through fermentation. The microbiota is responsible for up to 70% of the host’s daily energy requirement, and alterations in its composition can drastically influence food digestion and methane emissions (Cholewińska et al., 2021).

Forage-rich diets (high fiber content) favor the growth of fibrolytic bacteria, such as *Fibrobacter succinogenes*, *Ruminococcus flavefaciens*, and *Ruminococcus albus*, which act in the degradation of plant cell walls (Fernando et al., 2010; Henderson et al., 2015). On the other hand, high-concentrate diets (rich in starch) promote a greater abundance of amylolytic bacteria, such as *Streptococcus bovis* and *Prevotella* spp., while also reducing ruminal pH, which can compromise microbial diversity and favor disorders like acidosis (Petri et al., 2013; Plaizier et al., 2017). Abrupt changes in the forage-to-concentrate ratio (F:C) can lead to loss of microbial stability and reduced fermentative efficiency, representing a significant disruption of the evolutionarily established system. In this context, recent advances in molecular biology, such as next-generation sequencing, have enabled a deeper understanding of microbial diversity and metabolic pathways in the ruminal environment. In addition, to better characterize the functional potential of the microbial metagenome, approaches such as metabolomics, metatranscriptomics, and metaproteomics have been utilized (McCann et al., 2014; Du et al., 2024).

This review aims to evaluate how distant the diets used in current intensive cattle production systems are from ancestral diets based exclusively on forages, exploring the differences in ruminal microbiota between animals fed high-concentrate versus high-forage diets, characterizing the typical microbial profiles of each condition, and discussing the dynamics between beneficial and potentially detrimental microorganisms.

## 2 Characteristics of the ruminal microbiota

The ruminal microbiota is a highly diversified and densely populated ecosystem, composed of bacteria, protozoa, fungi, archaea, and viruses. To understand the impact of modern intensive systems, it is fundamental to contrast the ruminal microbiota of animals in systems based exclusively on forages (representing the ancestral evolutionary state) with that found in intensive production systems with high concentrate inclusion.

In animals maintained exclusively on pasture or forage-based diets, the ruminal microbiota presents characteristics that reflect millions of years of coevolution. These systems maintain high microbial diversity, a predominance of microorganisms specialized in fiber degradation, stable ruminal pH (6.5–7.0), and slow, continuous fermentation. In contrast, in modern intensive production systems, the high concentrate inclusion promotes dramatic alterations in this ecosystem (Squizatti et al., 2023), resulting in reduced microbial diversity (Pinto et al., 2020), predominance of amylolytic microorganisms over fibrolytic species (Pinto et al., 2023), reduced ruminal pH (Pinto et al., 2023; Pacheco et al., 2023), and rapid and intense fermentation. These shifts negatively affect pH-sensitive cellulolytic bacteria such as *Fibrobacter succinogenes* and holotrich protozoa, while favoring amylolytic populations like *Entodinium* (Pinto et al., 2023). This transition represents a critical period that demands specific adaptation protocols to prevent metabolic disorders (Rigueiro et al., 2021; Silvestre et al., 2023; Pinto and Millen, 2018).

Weimer (2015) describes the ruminal microbial community as redundant, resilient, and dependent on host individuality. Indeed, in the rumen, different microbial taxa are involved in the degradation of the same substrate, competing with each other for its utilization. This redundancy is not yet fully understood but may be involved in carbohydrate digestion (Gruninger et al., 2019). This hypothesis is reinforced by other studies (Taxis et al., 2015; Weimer, 2015), which show that alterations in microbial community composition do not always result in substantial changes in fermentation parameters, such as pH and SCFA concentration (Taxis et al., 2015). The redundancy of the ruminal microbiota also reflects the system’s resilience: some studies demonstrated the ability of rumen microorganisms to restore their structure after suffering different perturbations, such as temperature variations or alterations in ruminal pH (Weimer, 2015). Furthermore, other studies have reported a strong correlation between the productive efficiency of ruminants—including feed conversion, average daily gain, average daily intake, total weight gain, residual feed intake, milk production and quality—and ruminal microbial profiles in both beef and dairy cattle (Liu et al., 2022; Wang and Guan, 2022).

### 2.1 Bacteria

Bacteria are the most abundant (10^11^ cells/mL) and metabolically active microorganisms in the rumen. They possess numerous enzymatic activities and can be classified based on their preferred substrates (cellulolytic, amylolytic, proteolytic, lipolytic) and fermentation products. The dominant phyla include Firmicutes and Bacteroidetes, which together represent the majority of the bacterial community. Other important phyla are Proteobacteria, Actinobacteria, and Spirochaetes (Gruninger et al., 2019). Recent studies have demonstrated that the rumen presents a “core bacterial microbiome,” which is present in ruminants worldwide and includes members of different taxa, such as *Prevotella* spp., *Butyrivibrio* spp., *Ruminococcus* spp., members of the Ruminococcaceae and Lachnospiraceae families, and the Clostridiales and Bacteroidales orders (Henderson et al., 2015; Weimer, 2015).

The Clostridia class belongs to the Firmicutes phylum and is mainly involved in protein degradation. Key fibrolytic genera include *Fibrobacter succinogenes*, *Ruminococcus flavefaciens*, and *Butyrivibrio fibrisolvens*, which degrade cellulose, xylans, and pectins. However, recent metagenomic approaches have identified numerous additional bacterial taxa with fibrolytic, proteolytic, and other metabolic capabilities, many of which are highly sensitive to dietary changes and F:C ratio (Wang et al., 2019). Additionally, *Anaerovibrio lypolitica* decomposes fat and *Megasphaera elsdenii* utilizes glucose and lactate (Cabral and Weimer, 2024). The Bacilli class degrades proteins by secreting proteolytic enzymes (Palmonari et al., 2024). Another important phylum is Bacteroidetes, a group of gram-negative bacteria that includes the genus *Prevotella*. *Prevotella bryantii* and *Prevotella ruminicola* belong to this genus, whose many catabolic activities include assisting cellulolytic microorganisms in plant cell wall degradation through synergistic action with cellulolytic bacteria (Cholewińska et al., 2021). Bacteroidetes are also characterized by their ability to synthesize different vitamins, such as B1, B2, B7, B9, and B12. These vitamins are important for various functions in the rumen, such as amino acid, lipid, and carbohydrate metabolism, and act as cofactors in essential proteins that sustain cellular function in the ruminal microbial community and also for the host (Hernández et al., 2014). Cobalt is required for vitamin B12 synthesis by ruminal microorganisms, which acts as a coenzyme for methylmalonyl-CoA mutase, an essential enzyme in the conversion of propionate to glucose via the succinate pathway (González-Montaña et al., 2020; Girard and Duplessis, 2023).

As we will see later in this review, the Firmicutes and Bacteroidetes phyla are directly influenced by diet composition. The Firmicutes phylum tends to be more abundant in forage-rich diets, as many of its representatives possess the ability to degrade fibers and produce SCFA, such as acetate and butyrate (Clemmons et al., 2019; Gruninger et al., 2019; De Mulder et al., 2018). On the other hand, the Bacteroidetes phylum generally increases its relative abundance in diets with greater concentrate inclusion, due to its specialization in the degradation of non-fibrous polysaccharides and starches (Clemmons et al., 2019; Petri et al., 2013; De Mulder et al., 2018). Furthermore, high-forage diets usually favor greater bacterial diversity in the rumen, promoting a more stable and functionally diversified microbial ecosystem (Henderson et al., 2015). At a finer functional level, distinct families and genera within these phyla exhibit specialized enzymatic systems that reflect their dietary niches. Members of the Ruminococcaceae family within the Firmicutes phylum are key degraders of structural carbohydrates, secreting cellulases and xylanases that enable the breakdown of cellulose and hemicellulose (Flint et al., 2008; Gruninger et al., 2014; Huws et al., 2018). In contrast, genera such as *Prevotella* within the Bacteroidetes phylum are highly adapted to the utilization of non-fiber carbohydrates, producing amylases and glycosidases that facilitate the rapid fermentation of starches and soluble polysaccharides (Purushe et al., 2010; Stewart et al., 2018; Accetto and Avguštin, 2019). These functional differences help explain the contrasting responses of Firmicutes and Bacteroidetes to changes in the F:C ratio (Henderson et al., 2015; Gruninger et al., 2019).

Thus, the transition from diets based predominantly on forages to intensive systems with high concentrate content represents one of the most striking changes in ruminant nutrition. This transition is not limited to merely an adjustment in the profile of substrates available for fermentation but triggers a series of consequences on microbiota balance, ruminal health, and productive efficiency. From a microbiological perspective, this dietary change drastically alters the composition and function of the ruminal microbiota. In high-forage diets, fibrolytic bacteria such as *Fibrobacter succinogenes* and *Ruminococcus albus*/*Flavefaciens* predominate, which depend on plant cell wall degradation (Van Soest, 1994; Russell and Rychlik, 2001). In high-concentrate diets, there is a relative increase in amylolytic bacteria, such as *Streptococcus bovis* and *Prevotella* spp., in addition to the proliferation of lactate-utilizing bacteria, such as *Selenomonas ruminantium* and *Megasphaera elsdenii*, fundamental for preventing lactate accumulation (Morgavi et al., 2013; Petri et al., 2013). This functional substitution affects the nature of the metabolites produced, with a higher proportion of propionate and butyrate at the expense of acetate, altering the energy balance available to the host (Bannink et al., 2008; Huws et al., 2018).

**Figure 1:**
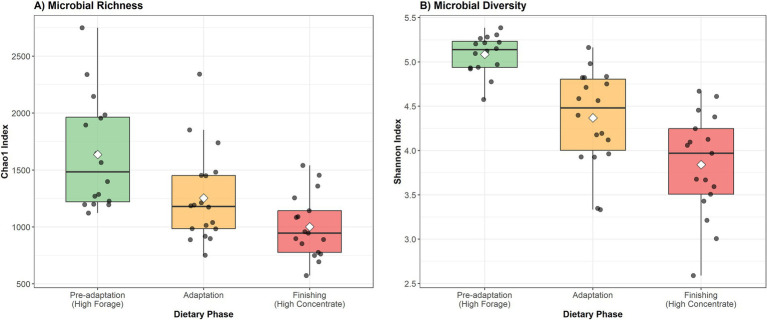
Changes in rumen microbial α-diversity at different dietary stages (Data Source: Pinto et al., 2020). Alpha diversity of the ruminal microbiota was evaluated in six cannulated Nellore bulls using 16S rRNA gene sequencing (V4 region) in a replicated 3 × 3 Latin square design. Animals were subjected to three dietary stages differing in forage-to-concentrate (F:C) ratio: pre-adaptation (high forage; F:C = 80:20), adaptation (transitional; F:C = 50:50), and finishing (high concentrate; F:C = 20:80). **(A)** Microbial richness estimated by the Chao1 index decreased by 39% from pre-adaptation (1,637 ± 491) to finishing (1,002 ± 276) (Kruskal–Wallis: *χ*^2^ = 17.18, *p* < 0.001). **(B)** Microbial diversity estimated by the Shannon index decreased by 24% from pre-adaptation (5.09 ± 0.22) to finishing (3.84 ± 0.58) (Kruskal–Wallis: *χ*^2^ = 30.38, *p* < 0.001). Boxplots represent the median (central line), interquartile range (box), and whiskers (1.5 × IQR). Individual samples are shown as gray dots, and white diamonds indicate group means. Error estimates are expressed as standard deviation. Overall, forage-rich diets support greater microbial diversity and richness, contributing to a more complex and stable ruminal ecosystem, whereas concentrate-rich diets reduce community complexity.

To illustrate the impact of dietary composition on ruminal microbial diversity, we reanalyzed data from Pinto et al. (2020), who evaluated the ruminal microbiota of Nellore cattle during dietary transition from high-forage to high-concentrate diets (Figure 1). Using 16S rRNA gene sequencing, microbial richness (Chao1 index) and diversity (Shannon index) were significantly higher in the high-forage diet compared to the high-concentrate diet, with Chao1 decreasing by 39% (from 1,637 ± 491 to 1,002 ± 276, *p* < 0.001) and Shannon diversity decreasing by 24% (from 5.09 ± 0.22 to 3.84 ± 0.58, *p* < 0.001). The adaptation phase showed intermediate values, reflecting the transitional nature of the diet. This shift toward a less diverse microbial community may increase susceptibility to metabolic disorders such as ruminal acidosis (Plaizier et al., 2008; Khafipour et al., 2009). However, modern feedlot management practices have successfully mitigated these risks through strategic interventions, including balanced diet formulation, gradual adaptation protocols, appropriate grain processing methods, and the use of feed additives such as ionophores and buffers (Nagaraja and Titgemeyer, 2007; Monsalve and Millen, 2025). When properly managed, high-concentrate diets enable substantial improvements in animal performance and feed efficiency without compromising ruminal health, as evidenced by the widespread success of intensive beef production systems worldwide (Owens et al., 1998; Rigueiro et al., 2021; Rigueiro et al., 2023; Silvestre et al., 2023). Understanding the specific microbial populations that dominate under different dietary conditions is therefore essential for optimizing both productivity and animal welfare.

### 2.2 Archaea

Archaea (10^6^ cells/mL) are responsible for methane production through hydrogenotrophic, methylotrophic, or, to a lesser extent, acetoclastic pathways. *Methanobrevibacter gottschalkii* and *Methanobrevibacter ruminantium* are the most abundant archaea in the rumen (Gruninger et al., 2019). Most of them utilize CO_2_ and H_2_, produced by non-methanogenic microorganisms, in the methanogenesis process, with electron donors for hydrogen production being mainly carbohydrates, but also alcohols and organic acids. Thus, archaea prevent excessive production of ethanol and lactate during fermentation, using these compounds as H_2_ donors (Cholewińska et al., 2021).

This hydrogenotrophic methanogenesis, mainly carried out by the genus *Methanobrevibacter*, is crucial for preventing the accumulation of hydrogen and maintaining ruminal fermentation. However, this process represents a significant energy loss for the host and contributes to greenhouse gas emissions (Janssen and Kirs, 2008; Morgavi et al., 2010). Forage-rich diets generally promote greater methanogenesis due to the fermentative profile being more favorable to hydrogen production (Beauchemin et al., 2020).

Methanogenic archaea also suffer impact during diet transition. While forage diets sustain a more diverse methanogenic community, associated with hydrogen fermentation derived from fiber degradation, concentrate diets tend to reduce archaeal diversity and favor certain methanogenic species (Plaizier et al., 2017; Janssen and Kirs, 2008). This shift in community composition can modify hydrogen fluxes and reduce the availability of H_2_ for methane production, as the greater use of hydrogen for propionate production in high-concentrate diets decreases methane formation (Lin et al., 2023), with both environmental and productive implications.

### 2.3 Fungi

Fungi (10^3^–10^6^ zoospores/mL), although less abundant than bacteria, are crucial in the degradation of lignified fibers and their populations fluctuate as a function of diet. The phylum Neocallimastigomycota is the most dominant in ruminants and includes the genera: *Anaeromyces*, *Caecomyces*, *Cyllamyces*, *Neocallimastix*, *Orpinomyces*, and *Piromyces* (Cholewińska et al., 2021; Sanjorjo et al., 2023).

Anaerobic fungi participate in fiber degradation and produce a set of enzymes (cellulolytic, hemicellulolytic, glycolytic, and proteolytic) that allow the degradation of plant structural polymers and increase the surface area available for colonization by other microorganisms. This increase occurs due to physical penetration and rupture of plant tissue by fungi as they grow (Akin and Borneman, 1990; Hess et al., 2011). Anaerobic fungi, including members of the phylum Neocallimastigomycota and other fungal groups, have the ability to colonize and degrade lignified fibers, an important process in forage-rich diets (Orpin and Joblin, 1997; Gruninger et al., 2014). They physically fragment plant structures, exposing cellulose and hemicellulose for action by other microorganisms, promoting fiber digestibility.

Ruminal fungi are generally associated with forage-rich diets, and their abundance decreases with the addition of concentrates (Cholewińska et al., 2021; Gruninger et al., 2019). Thus, anaerobic fungi, important in initial fiber degradation through perforation of plant cell walls, lose competitive space in high-concentrate diets, with negative consequences on the digestibility of residual forages (Orpin and Joblin, 1997; Edwards et al., 2017). It is noteworthy to mention that most fungi species are pH sensitive, and the accumulation of SCFA in the rumen may negatively impact their ability to grow (Griffith et al., 2009).

### 2.4 Protozoa

Ruminal protozoa (10^5^–10^6^ cells/mL) belong to the genera *Isotricha*, *Dasytricha*, *Entodinium*, *Diplodinium*, *Endiplodinium*, and *Epidinium*. The role of protozoa in the ruminal ecosystem is somewhat controversial (Weimer, 2015; Cholewińska et al., 2021; Costa-Roura et al., 2022).

Ciliated protozoa, particularly from the Entodiniomorphida and Holotricha families, present heterogeneous responses as a function of diet transition. While forage-rich diets favor greater diversity and population density, starch-rich diets tend to reduce ciliate abundance, especially in situations of abrupt ruminal pH drop (Newbold et al., 2015). Notably, there is a differential sensitivity to this pH drop among protozoan groups: holotrichs, such as *Isotricha* and *Dasytricha*, are generally considered more sensitive to acidic conditions than entodiniomorphs, like *Entodinium* and *Diplodinium*, which are more tolerant of lower pH environments (Nagaraja and Titgemeyer, 2007). This reduction can compromise starch sequestration processes and fermentation modulation, affecting ruminal cross-feeding balance. The protozoan population also tends to decrease in response to rumen acidification and increased ruminal content passage rate (Morgavi et al., 2010; Weimer, 2015). However, some protozoan species may thrive depending on starch availability in the diet (Williams and Coleman, 1992).

Defaunation studies have demonstrated that protozoa are not essential for rumen function, but their absence can influence food digestion (Mizrahi and Jami, 2018). Protozoa can engulf starch granules in the rumen and compete with amylolytic bacteria for substrate, reducing starch fermentation rate and the risk of ruminal acidosis. Furthermore, ruminal ciliated protozoa are associated with methane production through epi- and endosymbiotic relationships with methanogens (Gruninger et al., 2019; Solomon et al., 2022). Additionally, ciliated protozoa play an ambiguous role in the rumen. On one hand, they help stabilize pH by consuming starch and controlling bacterial populations (Williams and Coleman, 1992). On the other hand, they host methanogenic archaea in symbiosis, increasing methane production (Finlay et al., 1994). Furthermore, they prey on beneficial bacteria, which can reduce microbial protein synthesis efficiency, negatively impacting animal performance under certain conditions (Newbold et al., 2015).

### 2.5 Viruses

Viruses, particularly bacteriophages (phages), are the most numerous biological entities in the rumen, with concentrations estimated at 10^7^–10^9^ virus-like particles (VLPs) per milliliter of ruminal fluid, vastly outnumbering their microbial hosts (Lobo and Faciola, 2021). Despite their abundance, the rumen virome remains one of the least understood components of this ecosystem, often referred to as “viral dark matter” because of the high proportion of sequences that do not match known viral genomes (Yan et al., 2023). The dominant viral families identified in the rumen belong to the order Caudovirales, which are tailed phages, comprising primarily Siphoviridae, Myoviridae, and Podoviridae (Gilbert et al., 2020).

The primary mechanism by which viruses influence the rumen microbiota is through a predator–prey dynamic. In the lytic cycle, phages infect and lyse their bacterial and archaeal hosts, a process that directly shapes microbial community structure and diversity. This top-down control prevents any single microbial species from becoming overly dominant, thereby maintaining ecosystem stability and promoting biodiversity (Anderson et al., 2017). This viral-mediated lysis also contributes to nutrient cycling within the rumen through the “viral shunt,” where organic matter from lysed cells is released back into the environment, becoming available for other microorganisms.

Beyond predation, viruses are powerful agents of horizontal gene transfer. Through the lysogenic cycle, phages can integrate their genetic material into a host’s genome as a prophage. These prophages can carry auxiliary metabolic genes (AMGs), which may confer novel functional capabilities to the host, such as enhanced carbohydrate degradation or vitamin synthesis (Sato et al., 2024). This “bottom-up” effect can alter the metabolic potential of the microbial community, directly impacting feed digestion efficiency and the profile of fermentation end-products. For instance, phages are known to infect key fibrolytic bacteria, such as *Fibrobacter* and *Ruminococcus*, as well as methanogenic archaea, like *Methanobrevibacter*. By modulating the populations and functions of these critical microbes, the rumen virome has significant, though still largely unquantified, effects on fiber digestion, methane production, and overall host productivity (Wu et al., 2024; Yan and Yu, 2024).

Diet composition also appears to modulate rumen phage–host dynamics by indirectly shaping bacterial growth rates and metabolic activity. Diet-driven shifts in substrate availability influence microbial turnover and ecological dominance, which in turn affect bacteriophage infection strategies and host specificity (Anderson et al., 2017; Gilbert et al., 2020). Under high-concentrate diets, the increased abundance and activity of rapidly fermenting and amylolytic bacteria may favor higher phage infection rates targeting these populations, whereas forage-rich diets tend to support phage communities more closely associated with fibrolytic bacteria and structurally complex substrates (Yan et al., 2023; Wu et al., 2024). This selective pressure imposed by diet-driven microbial restructuring suggests that bacteriophages may play an active role in the adaptive remodeling of the ruminal microbiota during dietary transitions, contributing to the stabilization or destabilization of cross-feeding networks through top-down control and auxiliary metabolic functions (Sato et al., 2024).

Taken together, the taxonomic composition and functional roles of bacteria, archaea, fungi, protozoa, and viruses described above provide the ecological and metabolic foundation of the ruminal ecosystem. These microorganisms do not act in isolation; rather, their interactions and functional redundancy determine the resilience, efficiency, and stability of ruminal fermentation. Dietary composition is the primary driver capable of reshaping these microbial interactions, selectively favoring specific functional guilds and altering cross-feeding networks. In this context, understanding how distinct dietary patterns modulate the structure and function of the ruminal microbiota is essential. The following section therefore focuses on comparing microbial community profiles and interaction mechanisms under forage-rich and concentrate-rich diets.

## 3 Ruminal microbiota profile in different diets

Diet plays a crucial role as a modulator of the ruminal microbiota, being able to influence its composition and activity in all phases of cattle growth. Each variation in the physical and chemical composition of the diet creates a specific niche that selects particular microorganisms (Gruninger et al., 2019). Newbold and Ramos-Morales (2020) summarized the main objectives of ruminal microbiome manipulation through diet: improving the nutritional composition of ruminant products, promoting animal health, preventing toxin accumulation, reducing pathogen development, decreasing the environmental impact of livestock (with reduction in greenhouse gas emissions), optimizing the production of SCFA and microbial protein, in addition to reducing ammonia production in the rumen.

The diet of adult ruminants is generally based on forages, which provide fibrous fractions (mainly pectins, cellulose, hemicellulose, and lignin), complemented with concentrates to balance energy, nitrogen, amino acid, mineral, and vitamin requirements. Alterations in the chemical composition of forages or the addition of concentrates to the diet can also modify the composition of the ruminal microbial community (Gruninger et al., 2019). A high forage content in the diet reduces the risk of ruminal acidosis and favors the growth of specific microorganisms, such as cellulolytics.

On the other hand, high-concentrate diets may decrease ruminal pH and negatively affect bacterial richness and diversity (Sanjorjo et al., 2023). The sensitivity of the rumen to acidification is drastically increased by microbiota disruption, also known as disbiosis. In forage-based systems, microbial diversity and cross-feeding networks provide multiple buffering and pH stabilization mechanisms. When these networks are broken, the rumen becomes highly susceptible to pH fluctuations, potentially developing acute or subacute ruminal acidosis, conditions practically nonexistent in animals maintained exclusively on pasture. Furthermore, the inclusion of plant secondary metabolites, such as tannins, in ruminant diets can act as a source of stress for ruminal microorganisms, while the administration of probiotics (yeasts) can increase the abundance of certain microbial strains (Costa-Roura et al., 2022).

Thus, the transition from forage-rich diets to those with greater concentrate inclusion is a critical point in ruminant nutrition, especially in intensive meat and milk production systems. This change implies not only a modification in the supply of fermentable substrates to the rumen, but also a profound restructuring of microbial interactions (cross-feeding), ruminal physiology, and animal metabolism. The consequences of this transition can be analyzed in two complementary dimensions: (i) negative effects, related to ruminal ecosystem disruption, acidosis risk, and loss of microbial diversity; and (ii) positive effects, associated with increased energy utilization efficiency and greater animal productivity, when nutritional management ensures balance between fermentation, absorption, and adaptation (Plaizier et al., 2008; Dijkstra et al., 2012; Henderson et al., 2015).

### 3.1 Ruminal cross-feeding and diet transition

**Figure 2:**
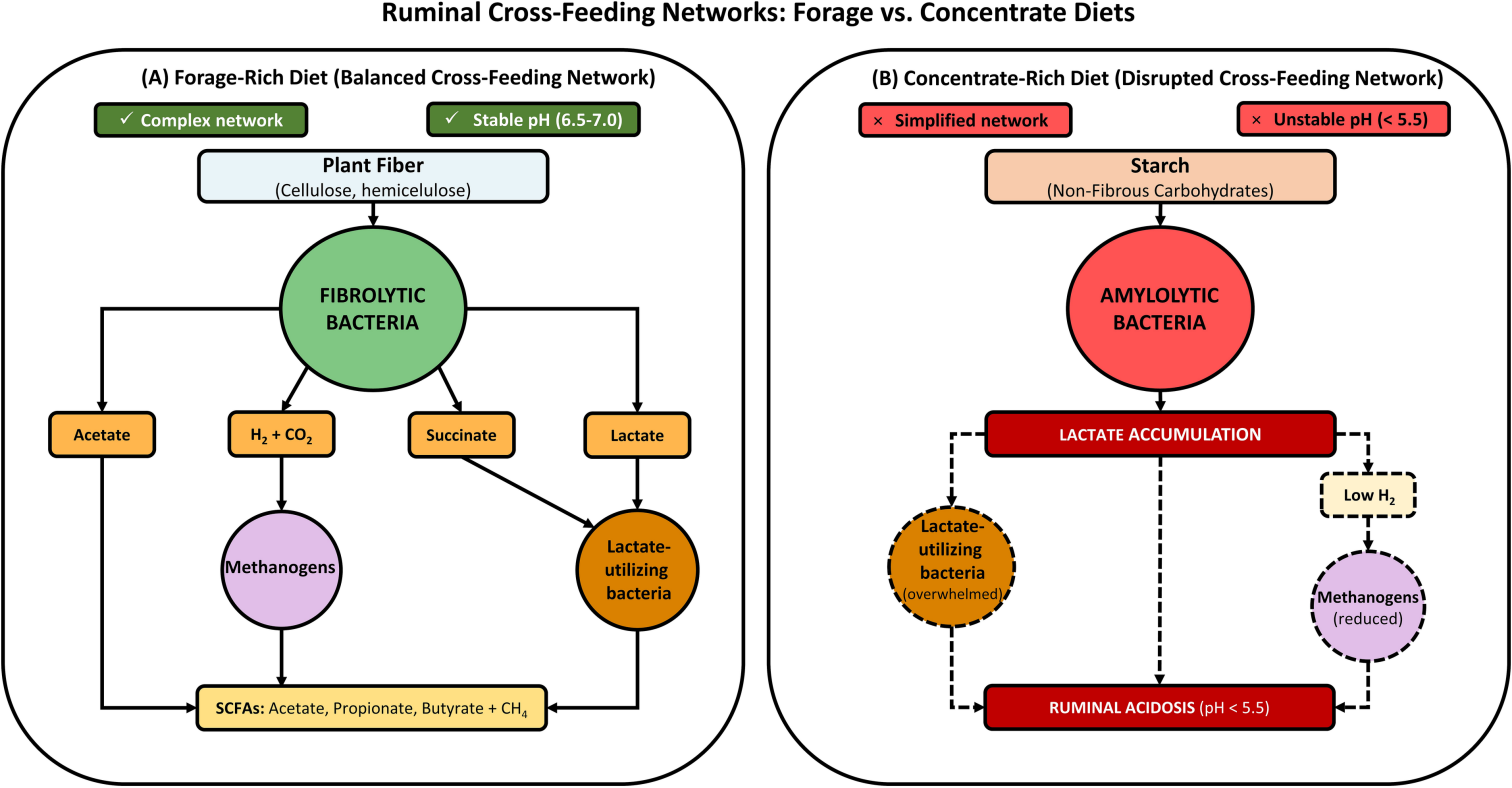
Conceptual representation of ruminal cross-feeding networks under forage-rich and concentrate-rich diets. **(A)** (Forage-rich diet—Balanced cross-feeding network): In forage-based diets, a complex and stable cross-feeding network is established. Fibrolytic bacteria degrade plant fiber into fermentative products such as acetate, succinate, and lactate, while also releasing gaseous substrates (H_2_ and CO_2_) as by-products of anaerobic fermentation. These metabolites are efficiently utilized by secondary fermenters: lactate- and succinate-utilizing bacteria convert these intermediates into propionate and butyrate, whereas methanogenic archaea consume H_2_ and CO_2_ to produce methane (CH_4_). This coordinated metabolic interaction maintains ruminal pH within the physiological range (6.5–7.0) and supports a balanced profile of short-chain fatty acids (SCFAs: acetate, propionate, and butyrate). **(B)** (Concentrate-rich diet—disrupted cross-feeding network): In concentrate-rich diets, rapid fermentation of starch and other non-fibrous carbohydrates by amylolytic bacteria leads to excessive lactate production, overwhelming the capacity of lactate-utilizing bacteria (indicated by dashed borders). Simultaneously, reduced fiber availability decreases the production of H_2_, limiting methanogenic activity (shown as reduced activity with dashed borders). Lactate accumulation results in a sharp decline in ruminal pH (<5.5), leading to metabolic imbalance and acidosis. Circles represent major microbial functional groups, including fibrolytic bacteria, amylolytic bacteria, lactate-utilizing bacteria, and methanogenic archaea. Rectangular boxes represent substrates, fermentation products, or metabolic outcomes within the ruminal ecosystem. Solid arrows indicate active metabolic pathways, whereas dashed arrows and dashed borders indicate reduced, overloaded, or destabilized processes under concentrate-rich dietary conditions.

Cross-feeding describes the exchanges of metabolites, electrons, and cofactors between microorganisms that allow the ruminal community to function as a highly interdependent consortium. In the rumen, this includes, above all, (i) interspecific hydrogen transfer (IHT) between H_2_ producers (e.g., cellulolytic bacteria, fungi, and ciliated protozoa) and H_2_ consumers (methanogenic archaea and fumarate/nitrate-reducing bacteria) (Greening et al., 2019; Ungerfeld, 2020), and (ii) the transfer of carbon intermediates (lactate, formate, succinate, ethanol, malate, amino acids, mono/oligosaccharides) between guilds that degrade plant polymers and guilds that ferment/convert intermediates (Solden et al., 2018). This view, now supported by high-resolution meta-omics techniques, shows that plant polysaccharide degradation is literally “orchestrated” by cross-feeding chains that connect Bacteria, Archaea, Fungi, and Protozoa (Solden et al., 2018) (Figure 2).

### 3.2 Cross-feeding in equilibrium state

In diets with high forage proportion, the fibrolytic consortium assumes a central role: bacteria such as *Ruminococcus*, *Fibrobacter*, and *Butyrivibrio*/*Pseudobutyrivibrio* act complementarily in fiber degradation, releasing sugars and oligosaccharides that serve as immediate substrate for secondary fermenters. This network is strengthened by anaerobic fungi (*Neocallimastigomycota*), which physically penetrate the plant wall, exposes new substrates, and secretes a broad-spectrum of CAZymes. Their hydrogenosomes produce H_2_ and formate, which are transferred to methanogenic archaea through interspecies hydrogen transfer (IHT) (Gruninger et al., 2014). Ciliated protozoa also contribute to preying on bacteria and restructuring the trophic web, as well as they accumulate starch and particles, helping to buffer fermentative peaks. They also release H_2_, which is utilized by symbiont archaea in ecto- or endosymbiotic relationships (Newbold et al., 2015). The result of this arrangement is a SCFA profile dominated by acetate and butyrate, efficient methanogenesis (maintaining low H_2_ partial pressure), and greater resilience to oscillations in fiber supply (Greening et al., 2019; Henderson et al., 2015).

The IHT constitutes the thermodynamic pillar of this equilibrium: by removing H_2_ via methanogenesis or alternative routes (such as fumarate reduction to succinate/propionate, or nitrate/nitrogen oxide reduction), where cellulolytic fermentation is favored. This increases ATP yield and fiber degradation rate (Ungerfeld, 2020). Evidence from co-cultivation and meta-transcriptomics confirms this mechanism, showing that hydrogenases modulate their expression according to H_2_ availability or removal—reinforcing the role of this gas as the central “redox currency” in the rumen (Greening et al., 2019).

### 3.3 What destabilizes cross-feeding?

The shift toward amylolytic and lactate-producing microorganisms during rapid concentrate inclusion creates a selective pressure that fundamentally alters ruminal ecosystem stability. When the production of fermentation end-products exceeds the ruminal buffering capacity, acidification of the environment occurs, triggering a cascade of ecological consequences that extend beyond simple compositional changes in the microbial community. The selective pressure caused by low pH reduces species richness and simplifies cross-feeding networks. Ciliated protozoa, important in starch fermentation modulation and protein recycling, have their population drastically reduced in more acidic environments (Williams and Coleman, 1992). Anaerobic fungi, essential in initial fiber fragmentation and structural sugar availability, also lose competitiveness. Consequently, microbial ecology becomes dominated by restricted and more resilient groups, increasing the risk of functional instability (Wang et al., 2023; Tapio et al., 2017). Within this acidified environment, specific metabolic guilds become particularly vulnerable to pH stress. Lactate-utilizing bacteria may be depleted at pH below 5.4, while lactate-producing species such as *Streptococcus bovis* and Lactobacillus spp. proliferate at pH below 5.6 (Owens et al., 1998; Nagaraja and Titgemeyer, 2007). This metabolic imbalance exacerbates lactate accumulation and further pH decline, creating a positive feedback loop that compromises ecosystem resilience (Plaizier et al., 2008).

The reduction in microbial richness and diversity can compromise system functionality and resilience, making it more susceptible to pathogen invasion (Fernando et al., 2010; Zhang et al., 2017). However, studies have shown that more efficient cows present lower microbial richness and diversity in the rumen (Shabat et al., 2016; Lopes et al., 2021). This finding suggests that high diversity is not always an essential condition for nutritional wellbeing, as long as animal requirements are met and the adaptation period to high-concentrate diets is respected. For example, Fernando et al. (2010) observed that the gradual inclusion of grains during the adaptation period increased bacterial richness and diversity due to the greater supply of available substrates and the establishment of new metabolic niches. Zhang et al. (2017) reported that cattle subjected to the limit-feeding strategy with high concentrate levels (up to 80%) showed altered ruminal fermentation and reduced bacterial diversity, although animal performance and nutritional requirements were maintained through controlled feeding management. However, as emphasized by Weimer (2015), bacterial functionality in the rumen is frequently redundant among different taxa, so predicting function based solely on microbial abundance can be imprecise. Thus, the discussion should focus on the balance between yield and rumen stability: although high-concentrate diets generally reduce microbial diversity—something that, in humans, would be associated with pathological imbalances—in well-managed and adapted ruminants this does not necessarily imply harm to health or welfare, as long as nutritional requirements are fully met and acidosis and inflammation prevention strategies are applied.

The first axis to collapse under high starch load is that of lactate. The phylum Bacteroidetes (especially the genus *Prevotella*) and some members of Firmicutes (such as *Streptococcus bovis* and *Lactobacillus* spp.) tend to increase in high-concentrate diets due to their ability to ferment rapidly digestible carbohydrates. However, the proportion between Firmicutes and Bacteroidetes can be complex and variable. Although Bacteroidetes are frequently associated with carbohydrate-rich diets, the pH drop in high-concentrate diets can favor the proliferation of certain acid-tolerant Firmicutes species, such as *Streptococcus bovis*, which are one of the major lactate producers. In many studies, the Firmicutes:Bacteroidetes ratio increases in high-concentrate diets, especially under acidosis conditions, reflecting a shift toward a microbial profile more adapted to acidic environments with high starch availability (Palmonari et al., 2024; Zhang et al., 2017).

Thus, *Streptococcus bovis* and Lactobacillus (rapid lactate producers) thrive at lower pH. Under normal ruminal conditions, this lactate is efficiently consumed by two main bacterial groups with distinct metabolic pathways: *Megasphaera elsdenii*, which converts lactate to propionate (and other short-chain fatty acids such as acetate, butyrate, and valerate) via the acrylate pathway (encoded by the lcdA gene for lactoyl-CoA dehydratase), and *Selenomonas ruminantium*, which produces propionate primarily via the succinate pathway (involving methylmalonyl-CoA decarboxylase, mmdA gene) (Counotte et al., 1981; Reichardt et al., 2014; Cabral and Weimer, 2024). The current consensus is that *M. elsdenii* is the major lactate user in high-grain diets, while *S. ruminantium* acts as a significant but secondary contributor (Cabral and Weimer, 2024). However, if the lactate-consuming capacity does not grow in proportion to the producing capacity, lactate accumulates, intensifies acidification, inhibits fibrolytic bacteria, and disrupts redox pathways (Owens et al., 1998).

This imbalance also affects propionate formation routes, which in high-concentrate diets depend on two main cross-feeding axes: (i) succinate → propionate pathway (mediated by Bacteroidetes and Selenomonadales) and (ii) lactate → propionate pathway (mediated by *Megasphaera*, via the acrylate pathway) (Counotte et al., 1981; Reichardt et al., 2014; Tapio et al., 2017; Desvignes et al., 2025). When well coupled, these routes compete with methanogenesis for H_2_/reduced electrons, reducing the CH_4_ fraction and increasing energy efficiency (through greater propionate production). In other words, greater propionate production, a SCFA that consumes hydrogen, can reduce the availability of this substrate for methanogenic archaea (Janssen, 2010). Simultaneously, the reduced pH associated with these diets can inhibit some methanogenic archaeal species, although others, more acid-tolerant, can adapt and remain active, such as *Methanobrevibacter boviskoreani*, capable of thriving at pH close to 5.5 (Lee et al., 2013). This alteration represents a disruption of biogeochemical cycles evolutionarily established in the rumen, by shifting hydrogen flow from methanogenic pathways to alternative routes such as propionate formation, with implications for energy efficiency and methane emissions. However, if pH drops excessively (below 5.5), the succinate pathway becomes less efficient, and although the acrylate pathway can partially compensate, the pH sensitivity of key lactate-utilizing bacteria (*M. elsdenii* and *S. ruminantium*) leads to lactate accumulation rather than succinate accumulation (Chen et al., 2019; Cabral and Weimer, 2024). Under these conditions, the metabolic network loses modularity and functional redundancy (Tapio et al., 2017).

Protozoa enter this scenario with an ambivalent role. On one hand, they can store starch and attenuate fermentative peaks; on the other, they maintain close symbioses with methanogens (ecto/endo), providing H_2_ and formate (Newbold et al., 2015). Thus, protozoa defaunation reduces CH_4_ (about 5–15%) and increases microbial protein flow to the intestine, but its effects on pH and fiber degradation vary according to context (Solomon et al., 2022). In high-concentrate diets, changes in protozoan structure and their associated archaea correlate with both CH_4_ emissions and the reorganization of trophic interactions (Newbold et al., 2015).

Anaerobic fungi also suffer impact. In diets containing high level of forage, they break the plant wall with rhizoids and release polymers of difficult access. However, at chronically reduced rumen pH and with lower effective fiber supply, their abundance and activity drop, decreasing synergies with fibrolytic bacteria and IHT with methanogens (Gruninger et al., 2014). In practice, this leads to less redundancy in fiber attack and greater dependence on amylolytic and rapid fermenting guilds (Solden et al., 2018).

Finally, even with relatively stable abundance of *Methanobrevibacter* (especially co-associated with protozoa), highly fermentable diets can reduce the relative participation of methanogenesis in the redox balance, due to greater electron drainage for propionate formation. Still, the physicochemical adhesion of methanogens to protozoa and particles (e.g., via adhesins) facilitates IHT, preserving methanogenesis even at moderately acidic pH—until the point where lactate/propionate coupling finally breaks it (Filomena et al., 2016).

### 3.4 Ruminal cross-feeding: balance and imbalance

Co-occurrence analyses and multi-kingdom networks show that, as the concentrate fraction grows, the ruminal community tends to present lower complexity, with fewer connections between modules (e.g., between fibrolytics and intermediate consumers) and greater fragility when faced with perturbations such as pH drop or oscillations in starch load (Tapio et al., 2017). On a global scale, surveys confirm that diet is the main determinant of community structure, more than the host itself (Henderson et al., 2015). Thus, the properties of the cross-feeding network are highly plastic and substrate-driven.

This equilibrium, however, does not depend only on the microbiota: it is also regulated by the host. From a physiological perspective, dietary transition is directly reflected on the ruminal epithelium. In high-forage diets, the epithelium presents shorter and sparser papillae, adequate for an environment of moderate SCFA production. The introduction of concentrates stimulates papillae hyperplasia and elongation, increasing absorptive capacity, especially of butyrate, which is the main inducer of epithelial cell differentiation (Dirksen et al., 1985; Steele et al., 2011). However, in situations of rapid or poorly managed transition, absorption may not keep pace with the SCFA production rate, favoring subclinical ruminal acidosis (SARA) and, in extreme cases, acute acidosis (Plaizier et al., 2008).

In this context, acid–base balance results from the interaction between SCFA/lactate production, H_2_ consumption, epithelial absorption, and buffering capacity (saliva). The epithelium adapts to more fermentable diets through hyperkeratosis, papillogenesis, and coordinated regulation of transporters/lipid metabolism (Steele et al., 2011; Shen et al., 2019). This increases SCFA absorption and helps stabilize pH, a condition in which pro-propionate cross-feeding is maintained and performance improves (Aschenbach et al., 2011). When adaptation is insufficient, or the fermentable load grows too rapidly, the spectrum of SARA (subacute ruminal acidosis) to acute acidosis is established (Plaizier et al., 2017), marked by the mismatch between acid production and removal and by the collapse of the lactate/propionate axis (Owens et al., 1998; Plaizier et al., 2012).

In summary, we can synthesize the states of the cross-feeding network into three scenarios:

- Stable resilient type (high-forage diet)—dense and modular network; efficient IHT (methanogenesis + alternatives); high fibrolytic redundancy; abundant fungi and protozoa; stable pH; metaproteomes reveal complete fiber → sugar → SCFA chains (Solden et al., 2018; Ungerfeld, 2020).
- Highly efficient coupled type (balanced concentrate diet with adequate adaptation)—well-synchronized succinate and lactate → propionate routes; lower relative CH_4_; higher propionate/efficiency; epithelium with high absorption capacity; protozoa and methanogens maintain IHT and avoid redox feedback, without excess lactate (Greening et al., 2019; Steele et al., 2011).
- Inefficient and imbalanced type (high-concentrate diet with rapid or inadequate transition)—considerable increase in lactate producers; insufficient consumers; pH drop; loss of fungi/fibrolytics; simplified networks; methanogenesis loses relative participation but may persist via protozoa; risk of SARA/acidosis (Owens et al., 1998; Plaizier et al., 2012).

Thus, the nutritional objective is not to “abolish” disruption, but to redirect it toward a new equilibrium in which cross-feeding favors propionate (performance) without breaking lactate ↔ propionate coupling and IHT. For this, the following are essential: gradual adaptation to concentrate; maintenance of physically effective NDF; stimulation of lactate/succinate consumers; and support for epithelial function (Aschenbach et al., 2011; Plaizier et al., 2017).

From a zootechnical perspective, dietary transition can represent both risk and opportunity. On one hand, there is the risk of metabolic disorders, loss of feed efficiency, and even performance decline in cases of inadequate adaptation (Nagaraja and Titgemeyer, 2007). On the other hand, when conducted in a controlled manner, concentrate inclusion increases propionate production, which acts as a direct gluconeogenic substrate, improving feed conversion efficiency, weight gain in beef cattle, and milk production in cows (Allen, 2000; Reynolds et al., 2003). This alteration in the fermentative profile reduces energy losses in the form of methane, since pathways directed toward propionate act as sinks for reducing equivalents (H_2_) that would otherwise be utilized by methanogenic archaea (Ungerfeld, 2020). Thus, high-concentrate diets, by privileging cross-feeding chains directed toward propionate (e.g., *Selenomonas ruminantium* and *Veillonella parvula* consuming lactate), can increase feed conversion efficiency and reduce the environmental footprint of production.

Therefore, the transition can be viewed as a process of displacement between two distinct physiological states: one of greater stability and resilience (forage-based) and another of greater metabolic and productive intensity (concentrate-based). Thus, the consequences of diet transition in ruminants should be interpreted as a balance between benefits and challenges. Intensification via concentrate inclusion, when well managed, expands productive potential and can optimize energy efficiency. However, this intensification also exposes the ruminal system to risks of fermentative imbalances and cross-feeding disruptions. Understanding these processes is crucial for designing nutritional strategies that reconcile performance, animal health, and sustainability.

## 4 Nutritional strategies to modulate ruminal microbiota and fermentation in high-concentrate diets

The transition from forage-based to high-concentrate feeds in modern ruminant production systems represents a profound ecological shift, disrupting the co-evolved stability of the ruminal microbiome. As established in the previous sections, this dietary change triggers a cascade of events, including a decrease in microbial diversity, a drop in ruminal pH, and a fundamental alteration of the cross-feeding networks that govern nutrient flow and energy partitioning (Fernando et al., 2010; Petri et al., 2013).

Studies have consistently demonstrated that this transition results in a shift from fiber-degrading microbial communities (dominated by cellulolytic taxa) to amylolytic communities optimized for fermenting starch and readily fermentable carbohydrates, with individual animals exhibiting variable adaptation rates to concentrate-rich diets (Huang et al., 2021; Wang et al., 2019). This microbial succession typically requires a 10–14 day adaptation period for the ecosystem to reorganize and establish a new equilibrium in response to altered dietary substrate availability.

Consequently, optimizing ruminal fermentation under these intensive conditions is not merely a matter of maximizing energy extraction but a complex challenge of maintaining metabolic homeostasis and preventing disorders such as subacute ruminal acidosis (SARA). This requires a multi-faceted approach that integrates foundational management practices with the strategic application of feed additives designed to selectively modulate microbial populations and their metabolic outputs.

### 4.1 The nutritional framework: establishing microbial resilience

Before considering specific additives, the foundation of ruminal modulation lies in fundamental nutritional management. The most critical of these is a well-managed dietary adaptation period. A gradual transition from forage to concentrate, typically lasting a minimum of 14–21 days, is essential to allow for the parallel adaptation of both the host and its microbiota (Brown et al., 2006; Rigueiro et al., 2021). This period allows for the proliferation of lactate-utilizing bacteria, such as *Megasphaera elsdenii* and *Selenomonas ruminantium*, before rapid lactate producers like *Streptococcus bovis* can cause a toxic accumulation. Simultaneously, the ruminal epithelium undergoes significant morphological changes, with papillae growing to increase the absorptive surface area by as much as 55–85%, a crucial adaptation for handling the increased production of SCFAs (Rigueiro et al., 2021; Dieho et al., 2016).

The choice of grain within the concentrate portion is also critical, as different grains have varying rates of starch fermentation. For example, wheat and barley starch are fermented more rapidly than corn starch, leading to a faster and more pronounced drop in ruminal pH, which can overwhelm the rumen’s buffering capacity and lactate-utilizing bacteria if not managed properly (Brown et al., 2006; Wu et al., 2024). The inclusion of barley in the diet, for instance, has been shown to significantly affect the relative abundance of key phyla such as Firmicutes, Proteobacteria, and Bacteroidetes, while also promoting fermentative genera like *Solibacillus* and *Lysinibacillus*, which are known to enhance feed fermentation (Wu et al., 2024).

Grain processing methods further modulate these effects. Steam flaking, for instance, gelatinizes the starch granules, making them more available for microbial digestion compared to dry rolling or grinding. While this can increase energy extraction, it also accelerates fermentation. Studies have shown that steam-flaked corn can lead to a lower ruminal pH compared to ground corn, but may also enhance microbial protein synthesis due to the increased availability of energy, as evidenced by higher urinary purine derivatives and protozoal populations (Eghtedari et al., 2024). This highlights a key trade-off in modern nutrition: maximizing energy availability without inducing metabolic distress. Finally, increasing the feeding frequency (e.g., from once to twice daily) can significantly buffer post-feeding pH drops, creating a more stable environment that prevents the boom-and-bust cycles of lactate production and supports a more consistent cross-feeding activity (Benchaar et al., 2020). These foundational strategies—adaptation, F:C ratio, grain type and processing, and feeding frequency—are the first and most powerful tools to steer the ruminal ecosystem toward a stable and efficient state on a high-energy diet. It is within this context that feed additives have been developed, not as a replacement for good management, but as targeted tools to address the specific metabolic bottlenecks that arise in these highly productive, yet inherently unstable, systems.

### 4.2 Feed additives as targeted tools for microbial modulation

Ionophores, such as monensin and lasalocid, act by selectively inhibiting Gram-positive bacteria, thereby restoring the balance between lactate producers and consumers. By disrupting the transmembrane ion gradients of species like *Streptococcus bovis*, monensin curtails rapid lactate production. This creates a competitive advantage for Gram-negative lactate utilizers, particularly *Megasphaera elsdenii*. Recent metagenomic studies have quantified this shift, showing that monensin can reduce the relative abundance of Streptococcus to less than 0.1% of the community while simultaneously increasing the proportion of *Megasphaera* (Weimer et al., 2010). Furthermore, longitudinal studies have demonstrated that monensin decreases the overall diversity of the rumen microbiome in the short term, with specific effects on bacterial amplicon sequence variants (ASVs) such as *Bacteroidales* (ASV19) and *Streptococcus infantarius* (ASV94) (Chai et al., 2024). This targeted modulation of the lactate cross-feeding network directly translates to improved fermentation efficiency, with a documented 15–20% increase in the propionate-to-acetate ratio (Marques and Cooke, 2021). However, concerns have been raised regarding microbial adaptation and the potential development of ionophore resistance following long-term use, which may reduce effectiveness over time and motivate alternative strategies such as additive rotation or combination approaches (Russell and Houlihan, 2003; Weimer et al., 2008).

Probiotics aim to directly reinforce key microbial populations. The administration of live cultures of *Megasphaera elsdenii* has proven effective in rapidly establishing a robust lactate-consuming capacity, particularly in newly adapted animals, mitigating the risk of acidosis (Cabral and Weimer, 2024; Habib et al., 2022). Studies have shown that preweaning supplementation with *M. elsdenii* not only improves rumen development and growth performance in calves (Mazon et al., 2025) but also reduces the incidence and severity of acidosis during the transition to solid feed (Muya et al., 2015). Yeast probiotics, most notably *Saccharomyces cerevisiae*, work through a complementary mechanism. By scavenging residual oxygen, they create a more strictly anaerobic environment that benefits both cellulolytic bacteria and lactate utilizers. Furthermore, they can stimulate the growth of *M. elsdenii* and *S. ruminantium*, enhancing the rumen’s overall capacity to handle lactate. Studies consistently show that yeast supplementation increases microbial diversity (as measured by Shannon and Chao1 indices) and stabilizes ruminal pH, reducing the duration of time spent below the critical threshold of 5.8 (Zhang et al., 2024; Wang et al., 2025). Recent research has also demonstrated that yeast supplementation can alter the relative abundance of key genera such as *Prevotella*, *Bacteroides*, *Clostridium*, *Ruminococcus*, and *Fibrobacter*, all of which play critical roles in ruminal fermentation (Xu et al., 2025).

Plant secondary metabolites, including saponins, tannins, and essential oils, represent valuable tools for stabilizing ruminal fermentation during dietary transitions from forage to concentrate-rich diets. Saponins reduce protozoal populations by disrupting cell membranes, which can moderate the rapid fermentation changes characteristic of high-concentrate diets and help maintain pH stability during the transition period (Kholif et al., 2023). Tannins exert multiple stabilizing effects: by complexing with dietary proteins, they slow fermentation rates and reduce the rapid accumulation of volatile fatty acids (VFAs) that typically occurs during concentrate introduction; this moderation of fermentation kinetics is particularly critical during the early stages of dietary transition when buffering capacity is overwhelmed (Jayanegara et al., 2012; Salami et al., 2018). Essential oils can selectively modulate microbial populations during transition, with certain blends showing efficacy in reducing lactate-producing species while supporting lactate-utilizing bacteria, thereby reducing acidosis risk during the shift to high-concentrate diets (Vakili et al., 2013; Nhara and Baloyi, 2025). The strategic use of these plant-derived compounds during dietary transitions helps maintain microbial community stability and ruminal pH homeostasis, reducing the incidence of subacute ruminal acidosis (SARA) that commonly accompanies abrupt dietary shifts (Hassan et al., 2020).

### 4.3 Combined nutritional strategies: synergies and practical limitations

The stabilization of ruminal fermentation under high-concentrate feeding systems increasingly depends on the integrated and synergistic combination of nutritional management strategies and feed additives, rather than the isolated application of single interventions. This approach recognizes that ruminal instability during dietary transitions arises from multiple, interconnected processes, including rapid carbohydrate fermentation, lactate accumulation, hydrogen imbalance, and reduced functional redundancy within microbial networks. Consequently, effective modulation strategies must simultaneously target complementary metabolic pathways.

Several additive combinations exemplify this principle. The association of ionophores with live yeast or yeast-derived products represents one of the most studied synergistic strategies. Ionophores selectively inhibit fast-growing, lactate-producing bacteria, thereby moderating fermentation rates, whereas yeast supplementation can stimulate lactate- and succinate-utilizing bacteria, improve redox balance, and enhance ruminal pH stability (Newbold et al., 1996; Chaucheyras-Durand et al., 2008). Similarly, the combined use of ionophores with different chemical structures, such as lasalocid and narasin, has been shown to influence fermentation pathways in distinct yet complementary ways, with narasin often shifting fermentation toward increased propionate production and improved energetic efficiency (Miszura et al., 2023).

Beyond ionophores and yeast, other additive combinations have gained attention as tools to mitigate the risks associated with high-concentrate diets. The use of buffering agents in combination with phytogenic compounds or plant secondary metabolites may help attenuate excessive acid production while modulating microbial activity and fermentation kinetics, thereby improving ruminal pH stability during dietary transitions (Beauchemin et al., 2008; Patra and Saxena, 2009; Hassan et al., 2020). Likewise, the integration of probiotics with substrates that support their metabolic niche, often referred to as synbiotic approaches, may enhance microbial persistence and functional activity within the rumen ecosystem (Chaucheyras-Durand and Fonty, 2002; Markowiak and Śliżewska, 2018). Collectively, these strategies highlight that additive combinations are most effective when they target distinct and complementary points of the ruminal metabolic network rather than exerting redundant effects.

Despite their potential, the practical application of additive combinations is constrained by several limitations. Animal-to-animal variation in additive responses remains substantial and is likely driven by differences in baseline microbiota composition, host genetics, prior nutritional history, and adaptive capacity (Chai et al., 2024). Furthermore, prolonged use of certain additives, particularly ionophores, raises concerns regarding microbial adaptation and reduced efficacy over time, reinforcing the need for long-term studies and management strategies such as additive rotation or the use of compounds with distinct but complementary mechanisms of action (Russell and Houlihan, 2003).

Overall, combined nutritional strategies offer a flexible and ecologically grounded framework to enhance ruminal resilience under intensive production systems. When integrated with appropriate adaptation periods and informed by an understanding of microbial functional interactions, additive combinations can contribute to more stable fermentation, improved feed efficiency, and reduced risk of metabolic disorders during the transition from forage- to concentrate-based diets.

## 5 Future perspectives and applications in intensive production

While multi-omics technologies have advanced our understanding of ruminal microbiota and cross-feeding interactions, relatively few studies have integrated these approaches specifically to predict and prevent cross-feeding destabilization during dietary transitions. Most applications focus on characterizing microbial shifts post-hoc rather than developing predictive models for early intervention. The current literature, as reviewed herein, demonstrates that cross-feeding is not merely a phenomenon of microbial competition or cooperation, but rather a critical determinant of energy efficiency and ruminal health during the transition from forage to concentrate diets. We propose that future research must prioritize the development of microbiota-informed nutritional strategies that actively preserve cross-feeding networks rather than simply mitigating their collapse through feed additives.

Looking forward, the continued advancement of multi-omics technologies is paving the way for a more precise and mechanistically informed approach to nutritional modulation. Metagenomics, metatranscriptomics, and metabolomics allow for an unprecedented view of the ruminal ecosystem, moving beyond simply cataloging species to understanding their functional activity in real time (Wang et al., 2023). This integrated perspective will enable the development of next-generation probiotics composed of synergistic microbial consortia and the formulation of diets based on individual microbial and metabolic profiles. In this context, maximizing ruminal fermentation in modern systems requires an integrated, ecological approach that begins with foundational management respecting the rumen’s physiological limits and is subsequently refined with targeted nutritional interventions capable of steering microbial networks toward greater efficiency and resilience.

The integrated application of metagenomics, metatranscriptomics, metaproteomics, and metabolomics offers unprecedented opportunities for real-time monitoring of cross-feeding dynamics during dietary transitions. However, we contend that current implementations of these technologies remain largely descriptive rather than predictive. To move beyond documentation of microbial shifts, the field must develop algorithms and models that can predict cross-feeding imbalances before they manifest as clinical acidosis or reduced performance. Early detection of these imbalances, coupled with targeted interventions (whether nutritional, probiotic, or genetic), could represent a paradigm shift in intensive production management.

The emerging evidence for heritability of ruminal microbiota characteristics presents a compelling opportunity for microbiome-informed genetic selection of animals inherently capable of maintaining efficient cross-feeding networks, even under high-concentrate feeding regimens. Recent studies indicate that host genetics can influence both microbial community structure and functional potential, thereby contributing to inter-animal variation in fermentation efficiency and metabolic resilience (Difford et al., 2018; Wallace et al., 2019). Importantly, this approach should not be viewed as a replacement for sound nutritional management, but rather as a complementary strategy. The synergistic integration of genetic selection for microbiota efficiency, precision nutrition informed by individual microbial profiles, and real-time monitoring via digital technologies could fundamentally transform intensive production systems, shifting management paradigms from reactive intervention to proactive prevention of microbiota destabilization.

Precision nutrition, when informed by individual microbiota composition and metabolic capacity, offers the potential to optimize concentrate inclusion while maintaining cross-feeding stability. We recognize, however, that the current implementation of precision nutrition remains largely theoretical in commercial settings. The development of cost-effective, on-farm tools for microbiota monitoring and real-time dietary adjustment is essential for translating this concept into practical application. Furthermore, we argue that precision nutrition must consider not only individual animal characteristics but also the temporal dynamics of microbiota adaptation during dietary transitions—a dimension that remains understudied.

In conclusion, we propose that the future of intensive ruminant production lies in the integration of microbiota-informed precision nutrition, genetic selection for microbiota efficiency, and digital monitoring technologies—all unified by a fundamental commitment to preserving cross-feeding stability during dietary transitions. This integrated approach has the potential to simultaneously improve feed efficiency, enhance animal health, reduce environmental impacts, and increase the sustainability of intensive production systems. However, achieving this vision requires substantial investment in research infrastructure, development of practical on-farm tools, and a paradigm shift in how the industry conceptualizes the role of the ruminal microbiota in animal production.

## 6 Final considerations

The ruminal microbiota is a complex and dynamic ecosystem, fundamental for ruminant nutrition and health. Diet, particularly the proportion between concentrate and forage, exerts a profound influence on the composition and activity of this microbial community. High-concentrate diets, although they may optimize energy and protein production, tend to reduce microbial diversity and richness, decrease ruminal pH, and favor the proliferation of microorganisms associated with metabolic disorders such as acidosis, laminitis, liver abscesses, and bloat. In contrast, high-forage diets promote a more diverse microbiota and a more stable ruminal environment, with predominance of fiber-degrading microorganisms, essential for ruminal health and efficient forage utilization.

The transition to high-concentrate diets therefore represents a nutritional paradox: on one hand, there are clear risks of microbial instability, loss of diversity, and harm to animal health; on the other hand, when well conducted, this transition allows greater energy efficiency, productive performance, and even reduction of methane emissions. The central challenge of ruminant nutrition is to balance these effects, promoting a stable, functional, and adapted ruminal environment in which cross-feeding is reorganized in favor of productivity without compromising animal health and welfare.

The transition from forage-rich to concentrate-based diets represents a critical juncture in ruminant production where microbiota stability must be actively managed. As discussed throughout this review, successful navigation of this transition requires an integrated approach that combines: (i) nutritional management through gradual diet adaptation, appropriate grain selection, and strategic use of feed additives (plant secondary metabolites, probiotics, ionophores) to stabilize fermentation and prevent pH decline; (ii) genetic selection for animals with inherent capacity to maintain efficient cross-feeding networks even under high-concentrate feeding; and (iii) real-time monitoring using digital tools and multi-omics technologies to detect early signs of microbiota destabilization and enable proactive interventions. Rather than viewing these approaches as separate strategies, their synergistic combination offers the most promising pathway to achieve both productivity and animal health during dietary transitions.

It is important to emphasize that in both strategies (high concentrate or high forage), it is possible to achieve high productivity, as long as ruminal fermentation is respected. This means that specific groups of microorganisms must develop and be capable of fermenting the available substrates. For this, the ruminal environment and conditions for fermentation must be carefully managed, ensuring nutritional wellbeing and the animal’s overall health. The key lies in understanding and optimizing the ruminal environment so that beneficial microorganisms thrive, regardless of the production objective.

In summary, we move beyond descriptive accounts of ruminal microbial composition to emphasize the central role of cross-feeding networks as regulators of fermentation efficiency and ruminal health during dietary transitions. We propose a conceptual framework in which the maintenance of microbial symbiotic balance under high-concentrate feeding emerges as a key determinant of ruminal health and production stability. By integrating microbial ecology, nutritional strategies, and host genetic potential, this framework provides a unified perspective to guide future research and applied interventions aimed at improving efficiency, resilience, and long-term sustainability in intensive ruminant production systems.
